# Microfluidic Impedance Biosensor Chips Using Sensing Layers Based on DNA-Based Self-Assembled Monolayers for Label-Free Detection of Proteins

**DOI:** 10.3390/bios11030080

**Published:** 2021-03-13

**Authors:** Khaled Alsabbagh, Tim Hornung, Achim Voigt, Sahba Sadir, Taleieh Rajabi, Kerstin Länge

**Affiliations:** 1 Institute of Microstructure Technology, Karlsruhe Institute of Technology, Hermann-von-Helmholtz-Platz 1, 76344 Eggenstein-Leopoldshafen, Germany; khaled.alsabbagh@icloud.com (K.A.); timhornung@posteo.de (T.H.); achim.voigt@kit.edu (A.V.); tala_rajabi@yahoo.de (T.R.); 2 Institute for Micro Process Engineering, Karlsruhe Institute of Technology, Hermann-von-Helmholtz-Platz 1, 76344 Eggenstein-Leopoldshafen, Germany; sadir.sahba@gmail.com

**Keywords:** biosensor, immunosensor, cardiac troponin I, single-strand DNA, electrochemical impedance spectroscopy, label-free, proteins, microfluidic chip, self-assembled monolayers

## Abstract

A microfluidic chip for electrochemical impedance spectroscopy (EIS) is presented as bio-sensor for label-free detection of proteins by using the example of cardiac troponin I. Troponin I is one of the most specific diagnostic serum biomarkers for myocardial infarction. The microfluidic impedance biosensor chip presented here consists of a microscope glass slide serving as base plate, sputtered electrodes, and a polydimethylsiloxane (PDMS) microchannel. Electrode functionalization protocols were developed considering a possible charge transfer through the sensing layer, in addition to analyte-specific binding by corresponding antibodies and reduction of nonspecific protein adsorption to prevent false-positive signals. Reagents tested for self-assembled monolayers (SAMs) on gold electrodes included thiolated hydrocarbons and thiolated oligonucleotides, where SAMs based on the latter showed a better performance. The corresponding antibody was covalently coupled on the SAM using carbodiimide chemistry. Sampling and measurement took only a few minutes. Application of a human serum albumin (HSA) sample, 1000 ng/mL, led to negligible impedance changes, while application of a troponin I sample, 1 ng/mL, led to a significant shift in the Nyquist plot. The results are promising regarding specific detection of clinically relevant concentrations of biomarkers, such as cardiac markers, with the newly developed microfluidic impedance biosensor chip.

## 1. Introduction

Label-free biosensors allow direct detection of analyte molecules and cells and, hence, offer a tool for fast detection of biomarkers and pathogens. Particularly electrochemical biosensors offer advantages here, as they can be fabricated cost-effectively, array-compatible, and customized in a comparatively easy way. Combining them with microfluidics results in efficient analytical devices, e.g., for biomarker detection in point-of-care applications, where clinically relevant protein concentrations are often in the range of a few ng/mL. In principle, a (micro-)fluidic channel can easily be made separately, e.g., from polydimethylsiloxane (PDMS), and then connected to the sensor unit. The requirements for biosensing layers include both the capability of analyte-specific binding and the minimization of nonspecific binding. The latter is particularly important for label-free bio-sensors to avoid false-positive results [1,2,3,4,5,6,7].

Label-free electrochemical biosensors include impedimetric biosensors which measure the impedance, i.e., the opposition presented to a current in an alternating current (AC) circuit when a voltage is applied. The impedance is a complex quantity, and a common graphical representation is the Nyquist plot. This is a frequency response plot, where the values of the real part are plotted on the x-axis and those of the imaginary part on the y-axis. An ideal Nyquist plot shows a semicircle resulting from the dominating, kinetically limited charge transfer through the electric double layer at the electrode. Binding of analyte molecules to the electrode will influence the charge transfer and, hence, result in a shift of the Nyquist plot. As a consequence, the opportunity for charge transfer is an additional requirement for sensing layers of impedance biosensors. At low frequencies, Nyquist plots may show straight lines with a slope of 45°. This is characteristic for diffusion limited processes and described by the Warburg impedance [8,9,10,11,12].

Electrodes of impedance biosensors are typically made of gold. A well-established procedure for the introduction of functional groups on this material is to use suitably substituted thiols forming self-assembled monolayers (SAMs). Thiols with aliphatic hydrocarbon spacers of sufficient chain length lead to well-defined and stable SAMs of high density. The brush-like structure of such layers makes it possible to effectively reduce nonspecific protein adsorption on the underlying gold surface. However, such SAMs may result in insulating layers, hindering the charge transfer required for the transduction principle of impedimetric biosensors. The use of aromatic hydrocarbons featuring delocalized π-electrons would be more beneficial for charge transfer processes, but nonspecific protein adsorption in the subsequent measurements may increase because of a reduced density of the layer. Conductive polymers would offer an alternative, but are often linked with coating procedures more complex than wet chemistry [7,12].

Thiolated single-strand DNA (ssDNA) oligomers, on the other hand, can be packed densely on the gold surface by wet chemistry methods similar to those of thiolated hydrocarbons. Coimmobilization of thiolated ssDNA with thiolated hydrocarbons may be recommended to improve the integrity of the brush-like structure. The negatively charged backbone of the DNA oligomers—resulting from the composition of alternating sugar (deoxyribose) and phosphate groups—promises lower initial impedance values and, hence, the possibility of charge transfer events [13,14]. With impedance sensors, immobilized ssDNA has been used directly as probe for DNA detection (hybridization). Similar to this, immobilized oligonucleotides have been designed as aptamers for protein detection. Furthermore, ssDNA has been hybridized with the complementary ssDNA carrying an analyte-specific capture molecule. A more flexible approach would be the hybridization of surface-bound ssDNA with complementary ssDNA carrying functional groups allowing the covalent coupling of any capture molecule. However, this has rarely been used on impedance electrodes so far [15,16,17,18]. 

As an example, for the applicability of DNA-based SAMs in impedimetric biosensors for protein detection, we used a cardiac biomarker. According to the World Health Organization (WHO), cardiovascular diseases (CVDs) are the leading cause of death worldwide. In 2016, 85% of the people who died of a CVD suffered a stroke or a heart attack. In 2015, 37% of people who died under the age of 70 due to noncommunicable diseases died from a CVD. One way to reduce these numbers is to increase the survival rate by starting the treatment as early as possible, which requires an early diagnosis [19,20]. Diagnosis criteria of a heart attack (also called myocardial infarction) include the detection of biomarkers in blood. Particularly, a concentration increase of cardiac troponins I and T (cTnI, cTnT) indicate myocardial necrosis with normal levels being <0.5–2.0 ng/mL (lab-specific) and <0.1 ng/mL, respectively, and a factor increase of up to 40-fold and 40–60-fold, respectively, in case of an infarction [21,22].

In this work, we introduce an own design for a microfluidic impedance biosensor chip. The base plate is a microscope glass slide carrying the gold electrodes, while the microfluidic channel is added as a PDMS component. Biosensing layers used thiol-SAMs based on both aromatic hydrocarbons and DNA, where the latter showed best performance regarding suppression of nonspecific binding of human serum albumin (HSA) and specific binding of the cardiac marker troponin I via the corresponding antibody.

## 2. Materials and Methods

### 2.1. Fabrication of the Microfluidic Impedance Biosensor Chip

#### 2.1.1. Base Plate and Electrode Sputtering

Standard microscope glass slides were used as base plates for the impedance biosensor chips. They were thoroughly cleaned by rinsing with a detergent solution and brushing with a toothbrush. After rinsing with water, they were sonicated with bidistilled water for 5 min using an ultrasonic cleaning bath. Finally, they were rinsed with filtered 2-propanol and blown dry with filtered nitrogen gas; the filter pore size was 0.2 µm in both cases.

A parylene C (poly(2-chloro-p-xylylene)) layer with a thickness of 0.1 µm was applied on the cleaned glass slides as adhesion layer (SCS Labcoter^®^ 1, PDS 2010, Specialty Coating Systems) [23]. Since the parylene C coating did not interfere with the later bonding of the PDMS microfluidic channel (see Section 2.1.2), it could be deposited on the entire surface of the microscope glass slides without the need of a mask or the removal of the coating outside the electrodes (see below). This made the coating process easy and convenient, which is why parylene C was preferred over the metal adhesion layers that were otherwise used for gold electrodes.

**Figure 1:**
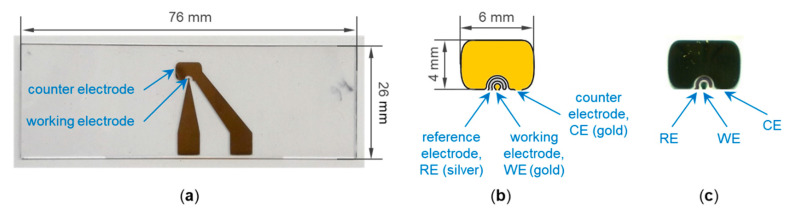
(**a**) Microscope glass slide with sputtered electrodes. (**b**) Detailed view of working and counter electrode, including a potential reference electrode. (**c**) Realized three-electrode setup sputtered on glass.

Working and counter electrodes made of gold were applied on the parylene C-coated glass slides by using a corresponding mask made of steel and a sputtering system (Balzers MED 010). The gold was sputtered at approximately 0.06 mbar and a current of 30 mA with the time set to 15 min, which led to a thickness in the range 15–35 nm. The diameter of the working electrode was 0.5 mm, corresponding to an area of 0.2 mm^2^. The ratio of the areas of working electrode to counter electrode was approximately 1:100. A microscope glass slide with sputtered electrodes is shown in Figure 1a. Conducting paths led from the electrodes to the edge of the glass slide, where they formed contact pads for the connection to the measurement setup (see Section 2.3.1). To keep things simple, a reference electrode was not included in this work; but it can be included, if required (Figure 1b,c).

#### 2.1.2. Microfluidic Channel Fabrication and Connecting

**Figure 2:**
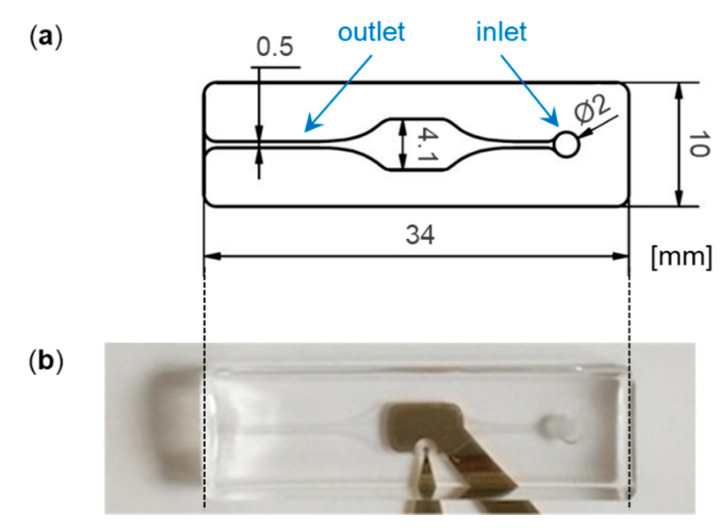
(**a**) Design of the microfluidic channel delivering samples across the electrodes. The channel height was 0.2 mm leading to a channel volume below 10 µL. (**b**) Microfluidic polydimethylsiloxane (PDMS) channel bonded on the impedance biosensor chip.

The microfluidic channel was formed from PDMS, the channel design is shown in Figure 2a. PDMS base and curing agent (Sylgard^TM^ 184, Dow) were mixed in a weight ratio of 10:1, following the manufacturer’s instructions. Air bubbles introduced by mixing the components were removed by evacuating the mixture in a vacuum. The bubble-free mixture was casted into a milled form made of polymethyl methacrylate (PMMA) and incubated at 70 °C for 2 h. To combine the PDMS channel with the impedance biosensor chip carrying the electrodes, both parts were plasma-activated and assembled with light pressure. During the plasma treatment, the electrodes were covered by impermeable poly-styrene pieces, as they had been functionalized with SAM compounds or antibody sensing layer before (see Section 2.2). A picture of the combined parts is shown in Figure 2b. 

### 2.2. Surface Functionalization

The deposition of the 0.05 or 10 µL drops of the reaction solutions described below was carried out manually by using microliter syringes and a magnifying lamp. Rinsing was performed with wash bottles. The antibody used in the following was monoclonal anti-troponin I, clone 1H11L19 (Fisher Scientific, Schwerte, Germany). 

#### 2.2.1. Antibody Adsorption

The electrodes were cleaned by plasma treatment. A 0.05 µL drop of an anti-troponin I solution, diluted with phosphate buffered saline (PBS) to 5 µg/mL, was deposited on the working electrode. After 4 h of incubation at 6 °C and rinsing with PBS, the chip was assembled with the PDMS microfluidic channel (see Section 2.1.2).

#### 2.2.2. Application of Thiol-SAMs with Hydrocarbon Spacer, Antibody Immobilization 

To test chemicals for use as SAM, the electrodes were cleaned by plasma treatment, and 10 µL of an ethanolic solution containing 50 mM 4-mercaptobenzoic acid, 1,4-benzenedithiol, or 6-mercapto-1-hexanol was deposited on both working and counter electrodes. After incubation overnight at ambient temperature, the electrodes were rinsed with ethanol and the chip assembled with the PDMS channel (see Section 2.1.2). 

A schematic representation of the antibody immobilization via SAM with hydrocarbon spacer and subsequent assay is given in Figure S1 in Supplementary Material. The electrodes were cleaned by plasma treatment, and a 0.05 µL drop containing 20 mM 4-mercaptobenzoic acid dissolved in ethanol was deposited on the working electrode. After 24 h of incubation at 6 °C, the chip was rinsed with ethanol. Another plasma cleaning step of the counter electrode was performed, during which the working electrode was covered with a piece of polystyrene. A 10 µL drop containing 20 mM 1,4-benzenedithiol dissolved in ethanol was deposited on the counter electrode and incubated overnight at 6 °C. After rinsing with ethanol and drying, 10 µL of a freshly prepared aqueous solution containing 0.05 M N-hydroxysuccinimide (NHS) and 0.2 M 1-ethyl-3-(3-dimethylaminopropyl)carbodiimide (EDC) was deposited on the electrodes and incubated for 30 min. The mixture was supposed to react only with the carboxyl groups of the 4-mercaptobenzoic acid SAM on the working electrode resulting in an active ester [24]. After rinsing with PBS, 0.05 µL of an anti-troponin I solution, diluted with PBS to 5 µg/mL, was deposited on the working electrode and incubated for 30 min. After rinsing with PBS, 0.05 µL of an aqueous solution of ethanolamine hydrochloride, 1 M, pH = 8.5, was deposited on the working electrode and incubated for 30 min to deactivate potentially available still reactive active ester groups [24]. Finally, the impedance biosensor chip was thoroughly rinsed with PBS, dried, and assembled with the PDMS microfluidic channel (see Section 2.1.2). 

#### 2.2.3. Application of Thiol-SAMs with DNA Spacer, Antibody Immobilization

Figure S2 shows the sequences of the ssDNA used below, including the schematic arrangement in the sensing layer. A schematic representation of the antibody immobilization via SAM with DNA spacer is given in Figure S3. Electrodes were cleaned by plasma treatment. SH-ssDNA (5′−thiol-C6-TTT TTT TTTTCC TGC GTC GTT TAA GGA AGT AC-3′, purchased from Metabion, Planegg, Germany) was coimmobilized with thiol compounds with hydrocarbon spacer for stabilization of the DNA-based SAM. The immobilization mixture contained either 0.025 mM SH-ssDNA and 15 mM 6-mercapto-1-hexanol or 0.033 mM SH-ssDNA and 13.3 mM 1,4-benzenedithiol dissolved in ethanol. A 0.05 µL drop of the immobilization mixture was deposited on the working electrode and incubated overnight at 6 °C. After rinsing with ethanol, the counter electrode was again cleaned by plasma treatment, while the working electrode was covered with impermeable polystyrene. A 10 µL drop containing 20 mM 1,4-benzenedithiol dissolved in ethanol was deposited on the counter electrode and incubated overnight at 6 °C. After that, 0.05 µL amino-ssDNA (5′-amino-C6-GTA CTT CCT TAA ACG ACG CAG G-3′, purchased from Metabion, Planegg, Germany), which was diluted with phosphate buffer to a concentration of 0.1 mM, was deposited onto the working electrode and incubated overnight at 6 °C. To convert the amino groups to carboxyl groups for antibody coupling, glutaric anhydride was dissolved in 8 M sodium hydroxide solution at a concentration of 0.5 mg/µL [25]; 10 µL of this solution was applied on the electrodes. The glutaric anhydride was supposed to react only with the amino groups of the functionalized working electrode. After 48 h of incubation at 6 °C, the chip was rinsed with bidistilled water. The following protocol of antibody coupling via EDC/NHS mixture and subsequent bonding of the PDMS channel was the same as described in the section before (Section 2.2.2).

### 2.3. Measurements with the Microfluidic Impedance Biosensor Chip

#### 2.3.1. Measurement Setup

Impedance measurements were performed with the IMPSPEC device from Meodat (Ilmenau, Germany), which was designed for fast and broadband impedance spectroscopy. The starting frequency was set to 5.895 Hz. The frequency was increased linearly by adding up frequency intervals of 5.895 Hz, until the final frequency of about 10 kHz was reached. The real part and the imaginary part of the impedance were displayed for each frequency. The impedance biosensor chip was connected to the IMPSPEC device cable by means of conductive silver and crocodile clamps. A peristaltic pump delivered the liquid samples through the PDMS channel and across the electrodes. 

#### 2.3.2. HSA Adsorption for Testing SAMs

PBS redox was prepared by adding potassium hexacyanoferrate(II) and potassium hexacyanoferrate(III) to PBS to a final concentration of 15 mM each. HSA was dissolved in PBS redox at concentrations of 0/1/10/100/1000 ng/mL, resulting in five HSA samples. Starting with a zero sample containing PBS redox only and corresponding to HSA, 0 ng/mL, the four other HSA samples were subsequently applied with increasing HSA concentration to the same impedance biosensor chip. Each sample was applied for 2 min at a flow rate of 0.04 mL/min. After sample application, the pump was stopped, and after 30 s, the impedance measurement was performed before the next sample was applied. After measuring the last HSA sample (1000 ng/mL), the impedance biosensor chip was disposed, and a new biosensor chip was used. 

#### 2.3.3. HSA Adsorption and Troponin I Assay

An impedance biosensor chip with freshly prepared antibody coating (see Section 2.2) and bonded PDMS microfluidic channel (see Section 2.1.2) was rinsed with PBS redox for 2 min at a flow rate of 0.04 mL/min. After switching off the pump and waiting for 30 s, the impedance of the antibody-coated biosensor chip was measured. After that, HSA dissolved at 1000 ng/mL in PBS was applied to the biosensor chip at a flow rate of 0.04 mL/min. Sampling time was 8 min if the antibody coating was performed without SAM (see Section 2.2.1) or 3 min if the antibody coating was performed via SAM (see Section 2.2.2 and Section 2.2.3). This was followed by PBS redox rinsing for 2 min at the same flow rate. The pump was stopped, and after 30 s, the effect of HSA on the respective biosensor impedance was measured. Finally, troponin I (Fisher Scientific, Schwerte, Germany) dissolved at 1 ng/mL in PBS was applied to the biosensor chip for 1 min at 0.04 mL/min, followed by PBS redox for 2 min at the same flow rate. The pump was stopped, and after 30 s, the impedance resulting from troponin I binding on the respective biosensor chip was measured. After measuring the troponin I sample, the impedance biosensor chip was disposed, and a new biosensor chip was used. 

## 3. Results and Discussion

### 3.1. Basic Performance of the Microfluidic Impedance Biosensor Chip

**Figure 3:**
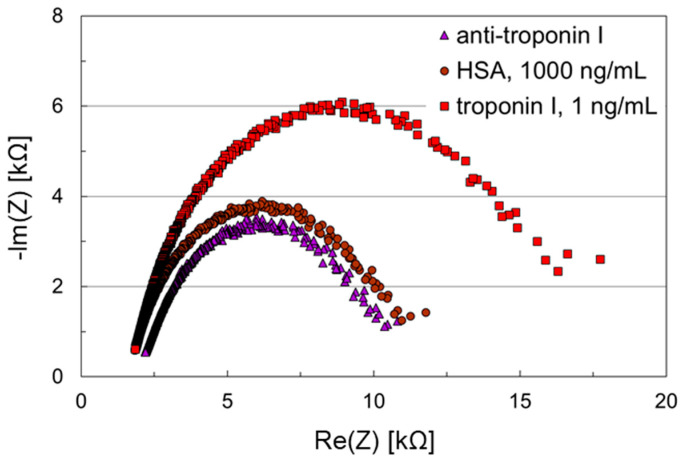
Nyquist plots of an impedance biosensor chip with no intermediary self-assembled mono-layer (SAM) for troponin I detection. Anti-troponin I was adsorbed on the working electrode, while the counter electrode remained uncoated. Samples containing human serum albumin (HSA), 1000 ng/mL, and troponin I, 1 ng/mL, were applied subsequently to the biosensor chip.

A preliminary experiment to test the performance of the microfluidic impedance bio-sensor chip was carried out by simply adsorbing the antibody on the working electrode, without the use of any SAM. While the working electrode was coated with anti-troponin I, the counter electrode remained uncoated. After application of HSA, 1000 ng/mL, for blocking remaining potentially accessible adsorption sites, troponin I, 1 ng/mL, was applied on the sensor surface. The results are shown in Figure 3.

The resulting Nyquist plots show almost ideal semicircles, i.e., charge transfer kinetics prevails the diffusion to the layer. Changes in the double layer, such as affinity binding to the surface, can be observed. Hence, the electrode design of the microfluidic impedance biosensor chip together with the measurement protocol with the redox buffer allows basic biosensor measurements. The recording of the impedances requires only a few seconds, the sampling time a few minutes. 

The initial impedance after antibody adsorption on the working electrode was 11 kΩ. It increased only slightly to 12 kΩ after HSA blocking, and the change in the complete Nyquist plot was negligible. After troponin I binding, however, the initial impedance increased to 18 kΩ, and a significant shift in the Nyquist plot was observed. This demonstrates that the chosen anti-troponin I is able to bind troponin I, while nonspecific HSA adsorption is blocked. Though this assay even worked with adsorbed antibody, antibody immobilization via SAM was to be performed in the following to show that the newly developed microfluidic impedance biosensor chip is also able to handle standard layer setups generally recommended for impedance biosensors. 

### 3.2. Testing Thiol-SAMs Based on Aliphatic and Aromatic Hydrocarbon Spacer

**Figure 4:**
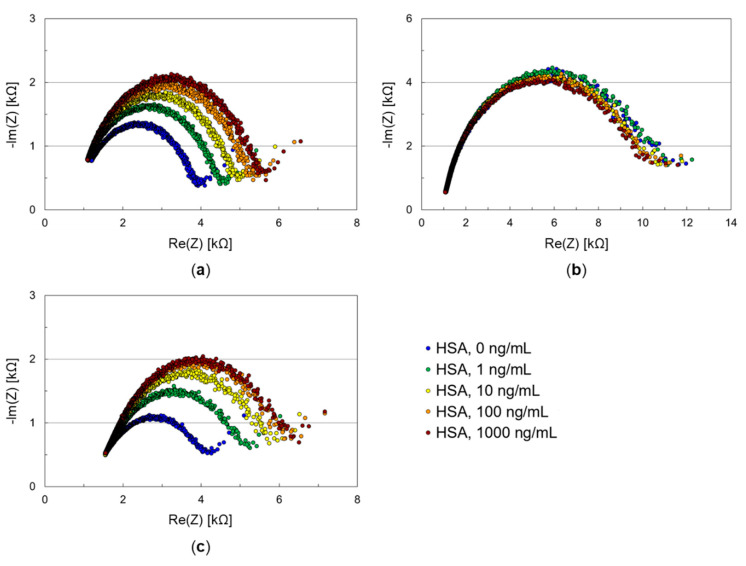
Nyquist plots of impedance biosensor chips for testing SAMs with HSA samples. Both working and counter electrode of such a chip were coated with (**a**) 4-mercaptobenzoic acid, (**b**) 1,4-benzenedithiol, or (**c**) 6-mercapto-1-hexanol. Samples containing HSA in increasing concentrations were applied subsequently to the biosensor chips.

SAMs on working and counter electrode are recommended to minimize nonspecific protein binding on the electrode surface, as this would lead to false positive signals, particularly in real samples with high concentrations of nonanalyte proteins. Furthermore, a suitable SAM on the working electrode needs to provide functional groups for antibody immobilization. To test the performance of potential SAMs with the microfluidic impedance biosensor chip, both working and counter electrode were coated with the respective thiol compound. After the SAM coating, samples containing increasing concentrations of HSA were successively applied on the electrodes. The results are shown in Figure 4.

Thiols with aromatic hydrocarbon spacers were chosen as they promise low initial impedance [12] and, hence, are beneficial for the charge transfer through the sensing layer. Both 4-mercaptobenzoic acid (Figure 4a) and 1,4-benzenedithiol (Figure 4b) resulted in SAMs yielding almost ideal semicircles except for a few frequencies at the beginning, confirming the possible charge transfer. 

The initial impedance of the 4-mercaptobenzoic acid SAM was 4.9 kΩ, after HSA adsorption it was 6.6 kΩ. i.e., the shielding against nonspecific adsorption was not perfect. However, 4-mercaptobenzoic acid offers a carboxyl group allowing further protein coupling by simple carbodiimide chemistry. The additional immobilization steps could increase the density of the sensing layer in a way that nonspecific HSA adsorption would be reduced to a greater extent. For that reason, experiments with 4-mercaptobenzoic acid as SAM on the working electrode were continued (see Section 3.3). 

The initial impedance of the 1,4-benzenedithiol SAM was 12 kΩ. This was higher than that obtained with 4-mercaptobenzoic acid, but it remained in this range after HSA sampling. Both 4-mercaptobenzoic acid and 1,4-benzenedithiol were applied in the same concentration (see Section 2.2.2), and their structure differs only in one functional group. However, 1,4-benzenedithiol has two thiol groups located *para* to each other which promotes the parallel or close to parallel orientation of the molecule on the gold surface [26,27], which is not the case for 4-mercaptobenzoic acid. Hence, a better surface coverage is obtained with 1,4-benzenedithiol, which makes it an ideal protection layer against unwanted protein adsorption.

Except for a few frequencies at the beginning, almost ideal semicircles in the Nyquist plot were also obtained for the 6-mercapto-1-hexanol SAM (Figure 4c), showing that the aliphatic hydrocarbon spacer is short enough to allow charge transfer through the sensing layer. The initial impedance was 5.2 kΩ, after HSA adsorption it was 7.3 kΩ. Hence, the results after HSA sampling were similar to those obtained with 4-mercaptobenzoic acid (Figure 4a) regarding both the initial impedances and the insufficient shielding against nonspecific protein adsorption. Better shielding might be achieved by using a thiol with a longer hydrocarbon chain, but this bears the risk of impeding the charge transfer. As 6-mercapto-1-hexanol in this study was not supposed to act as single SAM but as cocomponent in a DNA-based SAM (see Section 3.4), it is more important that the impedance values obtained with 6-mercapto-1-hexanol are not too high. This requirement is fulfilled, as shown above. 

### 3.3. Troponin I Assay Using Thiol-SAMs Based on Aromatic Hydrocarbon Spacer

**Figure 5:**
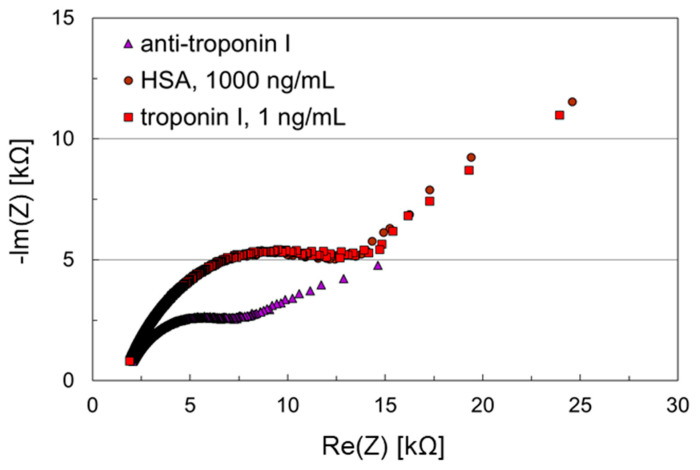
Nyquist plots of an impedance biosensor chip with aromatic hydrocarbon-based SAM for troponin I detection. The working electrode was coated with anti-troponin I, which was immobilized on a SAM consisting of 4-mercaptobenzoic acid. The counter electrode was coated with 1,4-benzenedithiol. Samples containing HSA, 1000 ng/mL, and troponin I, 1 ng/mL, were applied subsequently to the biosensor chip.

The working electrode was functionalized with 4-mercaptobenzoic acid acting as SAM with low impedance and providing functional groups for covalent antibody coupling. The counter electrode was coated with 1,4-benzenedithiol, as this showed the best shielding abilities against nonspecific protein adsorption compared with the other SAMs used here (see Figure 4). After antibody immobilization, samples containing HSA, 1000 ng/mL, and troponin I, 1 ng/mL, were applied successively to the microfluidic impedance biosensor chip. Figure 5 shows the results obtained with this assay.

The initial impedance after antibody coating was 15 kΩ. Antibody immobilization, however, did not reduce HSA adsorption as anticipated. Instead, after applying 1000 ng/mL HSA, the initial impedance almost doubled (27 kΩ) and remained in this range after troponin I, 1 ng/mL, was applied. Hence, troponin I detection was not possible with this impedance biosensor, at least not at that concentration. The functionality of the anti-troponin I used here was confirmed before (see Figure 3). It is unlikely that the antibody was harmed by the covalent coupling protocol, because this is a standard procedure for protein immobilization. Furthermore, the suitability of this approach is confirmed in the next section (Section 3.4). However, the accessibility of the antibody binding sites may be hindered by the comparatively large amount of nonspecifically adsorbed HSA.

A closer look at the Nyquist plots shows significantly increased linear ranges than obtained before. This is characteristic for the Warburg impedance and indicates that the diffusion of the charge carriers prevails over the charge transfer kinetics. Hence, in order to promote the charge transfer, another SAM was to be applied. SAMs with hydrocarbon spacers of larger chain lengths are favorable regarding prevention of nonspecific protein adsorption, but may result in high initial impedances hindering the charge transfer even more. As wet chemistry methods are preferred due to the less complex coating procedures, conductive polymers are not tested here. Instead, oligonucleotide spacers were tested, as DNA strands represent large chains with negative backbone, promising a lower initial impedance. 

### 3.4. Troponin I Assay Using Thiol-SAMs Based on DNA Spacer

**Figure 6:**
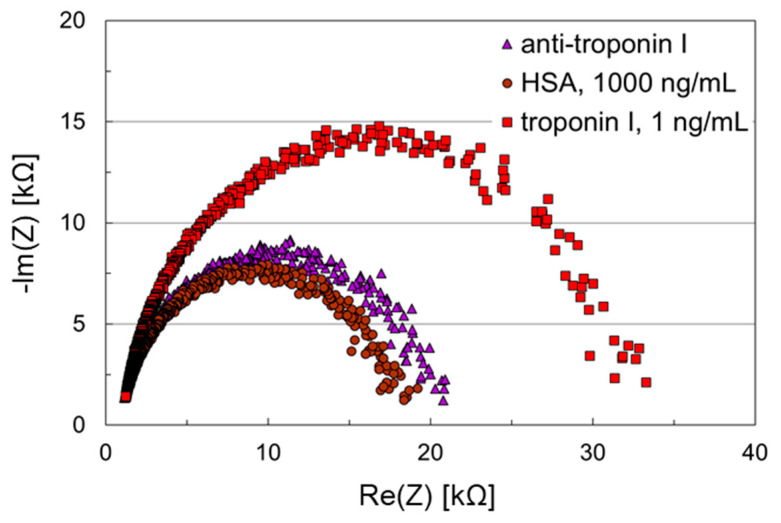
Nyquist plots of an impedance biosensor chip with DNA-based SAM for troponin I detection. The working electrode was coated with anti-troponin I, which was immobilized on a SAM consisting of thiolated DNA and 6-mercapto-1-hexanol. The counter electrode was coated with 1,4-benzenedithiol. Samples containing HSA, 1000 ng/mL, and troponin I, 1 ng/mL, were applied subsequently to the biosensor chip.

The SAM on the working electrode was formed by ssDNA carrying a thiol group and 6-mercapto-1-hexanol serving as coimmobilization agent. The latter was added to support the alignment of the ssDNA strands perpendicular to the surface so that the subsequent hybridization is not hindered [13,14]. After hybridization of the thiolated ssDNA with the complementary ssDNA carrying an amino group, functional groups for antibody immobilization were available (for details see Section 2.2.3). The counter electrode was coated with 1,4-benzenedithiol as before, because of the excellent shielding abilities. After antibody immobilization on the working electrode, HSA and troponin I samples were applied successively to the microfluidic impedance biosensor chip. The results are shown in Figure 6.

In contrast to the sensing layer based on a SAM with aromatic hydrocarbon spacer (Figure 5), the Nyquist plots obtained with the sensing layer using a DNA-based SAM showed ideal semicircles (Figure 6), indicating an enhanced charge transfer. After antibody coupling, the initial impedance was 21 kΩ. Application of the HSA sample, 1000 ng/mL, did not have a significant effect on the impedance values. Sampling with troponin I, 1 ng/mL, however, led to an initial impedance of 33 kΩ and a significant shift in the Nyquist plot. Hence, aside from kinetically controlled charge transfer, the DNA-based SAM allowed both shielding against nonspecific protein adsorption and detection of the cardiac marker corresponding to the antibody in a clinically relevant concentration. Furthermore, it is confirmed that the carbodiimide coupling procedure used here did not affect the integrity of the antibody. 

**Figure 7:**
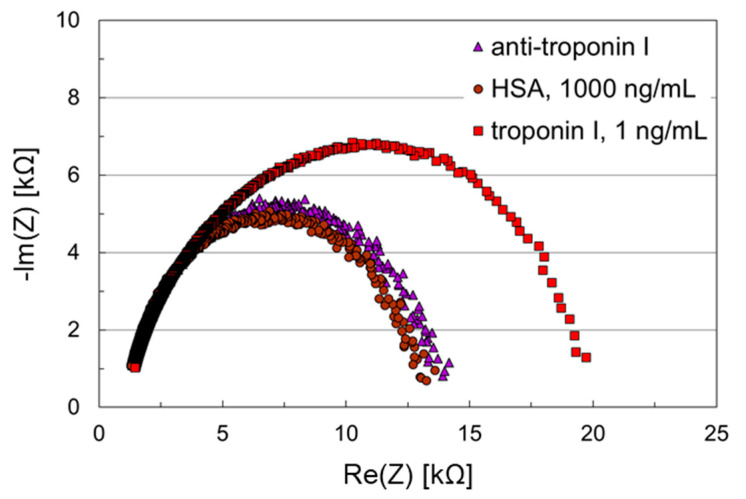
Nyquist plots of an impedance biosensor chip with DNA-based SAM for troponin I detection. The working electrode was coated with anti-troponin I, which was immobilized on a SAM consisting of thiolated DNA and 1,4-benzenedithiol. The counter electrode was coated with 1,4-benzenedithiol. Samples containing HSA, 1000 ng/mL, and troponin I, 1 ng/mL, were applied subsequently to the biosensor chip.

Finally, 1,4-benzenedithiol was used to replace 6-mercapto-1-hexanol as coimmobilization agent. This allowed the addition of potential benefits arising from the aromatic nature of the hydrocarbon spacer. Furthermore, this would simplify the coating procedure with regard to the use of fewer chemicals. The further immobilization and assay procedures remained the same. The results are shown in Figure 7.

Again, ideal semicircles were obtained in the Nyquist plot, confirming the improved charge-transfer in DNA-based sensing layers. The initial impedances decreased to 14 kΩ and remained in this range after application of the HSA sample, which led only to a negligible shift in the Nyquist plot. An initial impedance of 20 kΩ and a significant shift of the Nyquist plot was obtained by applying the troponin I sample. Though 1,4-benzenedithiol does not have the linear chain structure like 6-mercapto-1-hexanol, it still stabilizes the thiolated ssDNA strands in a way that the accessibility of the binding sites in the sensing layer is not hindered. As a result, the troponin I assay was also performed successfully with this DNA-based sensing layer. 

The next step would include tests in real sample media, such as blood and blood derivatives (serum, plasma) or saliva. Those media contain enzymes that may degrade the DNA-based sensing layer. It has to be tested whether this effect is notable already at a sampling time of 3 min (see Section 2.3.3). If so, the sampling time could easily be reduced. Furthermore, the addition of ethylenediaminetetraacetic acid (EDTA) to the sample or the use of EDTA plasma instead of serum could be considered. EDTA complexes Ca^2+^ and Mg^2+^ which are required as cofactors for the DNA-degrading enzymatic reaction. Hence, this complexation would reduce the interfering effects of the DNA-degrading enzymes, while troponin detection is still possible as is the detection of other proteins [28,29,30].

## 4. Conclusions

A microfluidic impedance biosensor chip was developed allowing the determination of 1 ng/mL cardiac biomarker troponin I. The use of thiols with DNA spacer showed excellent results regarding both reduction of nonspecific protein adsorption (HSA) and detection of low, clinically relevant concentrations of a biomarker (here: troponin I, 1 ng/mL). Sampling and measurement took only a few minutes, whereby the main part of the time was taken up by the sampling, which can easily be reduced by an optimized fluidic setup. The results were promising regarding a future cost-effective biosensor array chip for the rapid detection of clinically relevant biomarkers in real samples, such as serum and saliva. Future measurements will include measurements in real sample media to investigate the effect of potential DNA-degrading effects by enzymes in the sample media more closely. 

## Supplementary Materials

The following are available online https://www.mdpi.com/2079-6374/11/3/80/s1, Figure S1: Schematic representation of antibody immobilization on a thiol-SAM with aromatic hydrocarbon spacer (4-mercaptobenzoic acid), bonding of the PDMS microfluidic channel and subsequent assay with HSA blocking and troponin I sampling, Figure S2: Schematic representation of the single strand DNAs (ssDNAs) forming the thiol-SAM with DNA spacer for subsequent antibody immobilization (see Figure S3), Figure S3: Schematic representation of antibody immobilization on a thiol-SAM with DNA spacer (co-immobilization compound: 1,4-benzenedithol) and bonding of the PDMS microfluidic channel. The formation of peptide bonds in steps (5) and (6) is not included in this scheme for the sake of clarity. The subsequent assay with HSA blocking and troponin I sampling was performed as shown in Figure S1, steps (6) and (7).
