# Supplementary material for: Microfluidic Impedance Biosensor Chips Using Sensing Layers Based on DNA-Based Self-Assembled Monolayers for Label-Free Detection of Proteins
Source: Biosensors (Basel). 2021 Mar 13;11(3):80. doi: 10.3390/bios11030080 (PMC8001378; doi:10.3390/bios11030080)
Supplement: Supplementary file 1 [file biosensors-11-00080-s001.pdf]

Communication

Microfluidic Impedance Biosensor Chips Using Sensing Layers Based on DNA-Based Self-Assembled Monolayers for Label-Free Detection of Proteins

Khaled Alsabbagh[1], Tim Hornung[1], Achim Voigt[1], Sahba Sadir[2], Taleieh Rajabi[1], Kerstin Länge[1*]

Supplementary Material

Overview

Figure S1. Schematic representation of antibody immobilization on a thiol-SAM with aromatic hydrocarbon spacer (4-mercaptobenzoic acid), bonding of the PDMS microfluidic channel and subsequent assay with HSA blocking and troponin I sampling.

Figure S2. Schematic representation of the single strand DNAs (ssDNAs) forming the thiol-SAM with DNA spacer for subsequent antibody immobilization (see Figure S3).

Figure S3. Schematic representation of antibody immobilization on a thiol-SAM with DNA spacer (co-immobilization compound: 1,4-benzenedithol) and bonding of the PDMS microfluidic channel. The formation of peptide bonds in steps 5) and 6) is not included in this scheme for the sake of clarity. The subsequent assay with HSA blocking and troponin I sampling was performed as shown in Figure S1, steps 6) and 7).

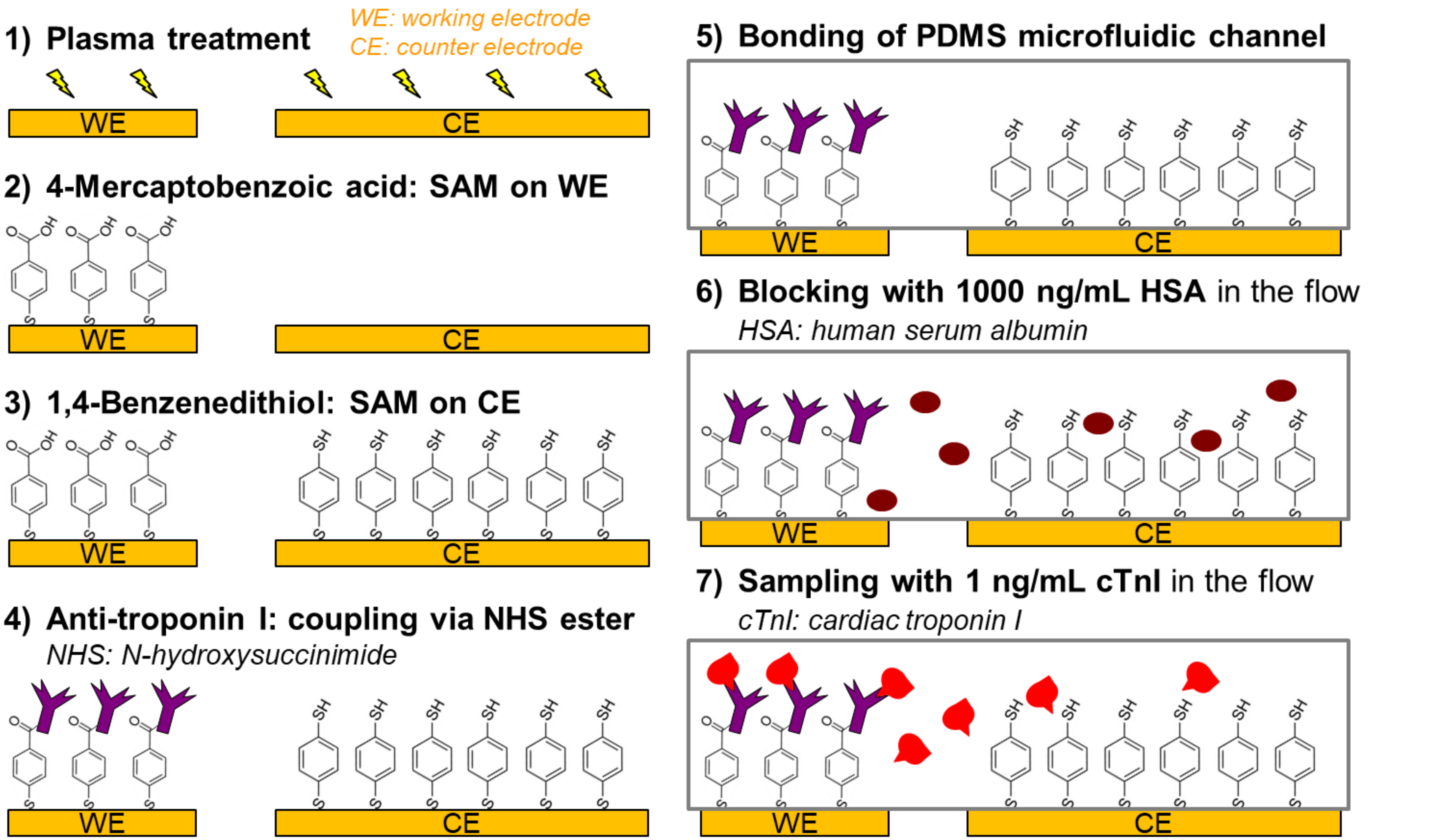

Figure S1. Schematic representation of antibody immobilization on a thiol-SAM with aromatic hydrocarbon spacer (4-mercaptobenzoic acid), bonding of the PDMS microfluidic channel and subsequent assay with HSA blocking and troponin I sampling.

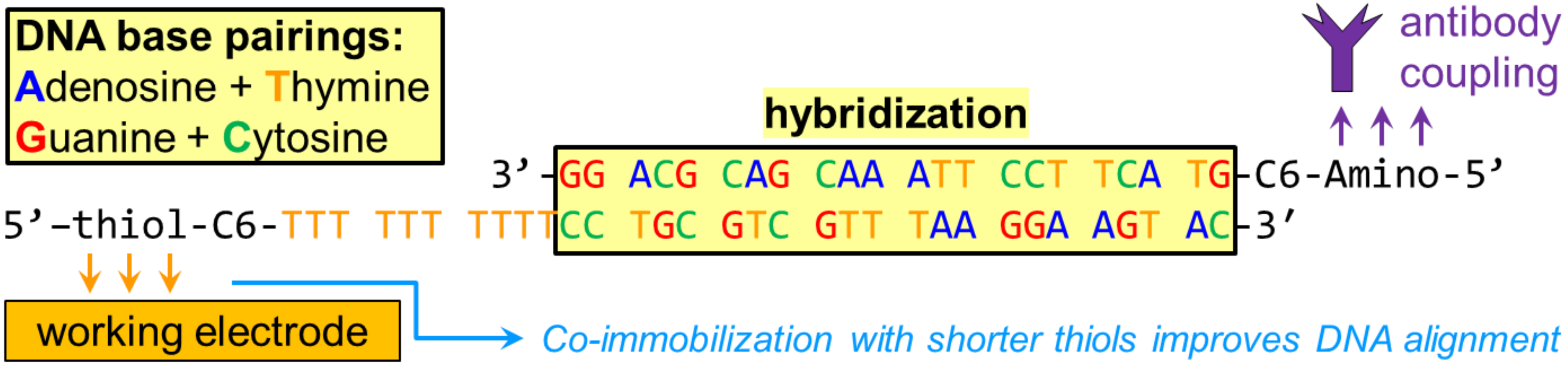

Figure S2. Schematic representation of the single strand DNAs (ssDNAs) forming the thiol-SAM with DNA spacer for subsequent antibody immobilization (see Figure S3).

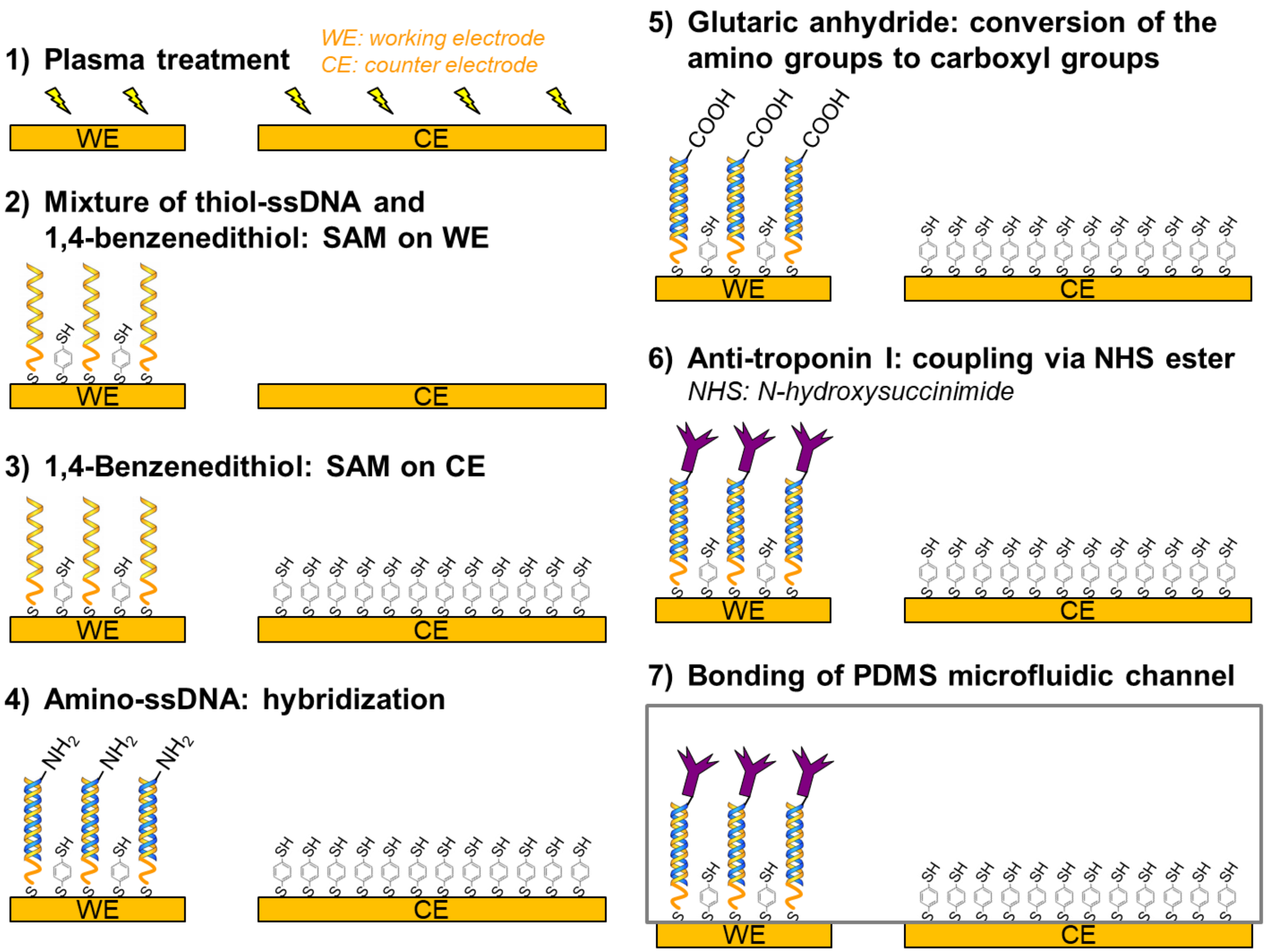

Figure S3. Schematic representation of antibody immobilization on a thiol-SAM with DNA spacer (co-immobilization compound: 1,4-benzenedithol) and bonding of the PDMS microfluidic channel. The formation of peptide bonds in steps 5) and 6) is not included in this scheme for the sake of clarity. The subsequent assay with HSA blocking and troponin I sampling was performed as shown in Figure S1, steps 6) and 7).
